# Thrombophilia genetic mutations and their relation to disease severity among patients with COVID-19

**DOI:** 10.1371/journal.pone.0296668

**Published:** 2024-03-20

**Authors:** Hend Moness, Suzan Omar Mousa, Sarah Omar Mousa, Nashwa Mohamed Adel, Reham Ali Ibrahim, Ebtesam Esmail Hassan, Nadia Ismail Abdelhameed, Dalia Abdelrahman Meshref, Noha M. Abdullah

**Affiliations:** 1 Clinical Pathology Department, Faculty of Medicine, Minia University, Minia, Egypt; 2 Pediatric Department, Faculty of Medicine, Minia University, Minia, Egypt; 3 Anesthesiology and Intensive Care Department, Faculty of Medicine, Minia University, Minia, Egypt; 4 Radio-Diagnoses at Minia University Hospital, Minia, Egypt; 5 Microbiology and Immunology Department, Faculty of Pharmacy, Minia University, Minia, Egypt; 6 Public Health and Preventive Medicine, Faculty of Medicine, Minia University, Minia, Egypt; 7 Internal Medicine, Faculty of Medicine, Minia University, Minia, Egypt; Kaohsuing Medical University Hospital, TAIWAN

## Abstract

**Objectives:**

Patients with COVID-19 infection appear to develop virus-induced hypercoagulability resulting in numerous thrombotic events. The aim of the present study was to determine the relationship between the thrombophilia genes mutations (prothrombin G20210A, factor V Leiden, and methyltetrahydrofolate reductase (MTHFR)) and the severity of COVID-19 patients.

**Design:**

Prospective cross-sectional study.

**Method:**

One hundred and forty patients (80 adults and 60 children) were included in the current study. They were divided into the severe COVID-19 group and the mild COVID-19 group, with each group comprising 40 adults and 30 children. The patients were assessed for FV R506Q, FV R2H1299R, MTHFR A1298C, MTHFR C677T, and prothrombin gene G20210A polymorphisms. CBC, D-dimer, renal and liver function tests, hs-CRP, ferritin, and LDH were also assessed. Thrombotic events were clinically and radiologically documented.

**Results:**

Severe COVID-19 cases were significantly more frequent to have a heterozygous mutation for all the studied genes compared to mild COVID-19 cases (p<0.05 for all). Being mutant to gene FV R506Q carried the highest risk of developing a severe disease course (p<0.0001). Patients with abnormally high D-dimer levels were significantly more frequent to be heterozygous for FV R506Q, FV R2H1299R, and prothrombin gene G20210A (p = 0.006, 0.007, and 0.02, respectively).

**Conclusion:**

We concluded that there is an evident relationship between severe COVID-19 and inherited thrombophilia. In the current study, FV R506Q gene mutation carried the highest risk of developing a severe COVID-19 disease course.

## Introduction

In December 2019, the China Health Authority and the World Health Organization (WHO) recognized numerous pneumonia cases with a high incidence of transmission among humans in Wuhan City. Many patients worked or lived around the local Huanan Seafood Wholesale Market [1]. This newly discovered virus was detected from the throat swab samples of patients. It was abbreviated as 2019-nCoV by the WHO [2], later renamed as severe acute respiratory syndrome coronavirus 2 (SARS-CoV-2) by the Coronavirus Study Group, and finally named coronavirus disease 2019 (COVID-19) by the WHO [3, 4]. Data published by the WHO on the 8th of October 2021 reported that the total number of people affected worldwide is 236,599,025, including 4,831,486 deaths [4]. The effect of SARS-CoV-2 is not limited to the respiratory system but extends to other systems, such as the gastrointestinal tract, cardiovascular, and central nervous systems [5]. Patients with COVID-19 infection appear to develop a virus-induced hypercoagulability resulting in numerous thrombotic events [6]. The mechanisms of COVID-19 associated thrombosis still unclear but may be due to systemic and endothelial inflammation which stimulate release of Willebrand antigen, fibrinogen, factor VIII activity51, immune-mediated damage by antiphospholipid antibodies and hypoxaemia-induced vascular occlusion [7–9]. Also, high levels of IL-1, IL-6, TNF-a, and thrombin facilitate clot formation in COVID-19 patients by activating platelets and inhibit the fibrinolytic processes by downregulation of the activated protein C. This imbalance between coagulants and anti-coagulants leading to pro-coagulation state in the form of pulmonary embolism (PE), deep-vein thrombosis, systemic arterial embolism, ischemia, and myocardial infarction [10, 11]. There was a suggestion of venous and a lesser extent of arterial thromboembolism, are common in patients with COVID-19 admitted in the intensive care unit [12]. Recent documentation revealed that the incidence of venous thrombosis (e.g., deep venous thrombosis) was higher in COVID-19 patients [13].

Thrombophilia results from genetic factors, acquired factors, or a mixture of both. Family-based approach recorded that more than 60% of venous thromboembolism susceptibility was attributable to genetic factors. The most common genetic factors are the prothrombin gene, factor V Leiden, and methylenetetrahydrofolate reductase (MTHFR) gene which are key polymorphic biomarkers of thrombophilia. Patients with multiple gene defects have a high risk of thrombosis [14, 15]. Factor V Leiden is the leading cause of protein C resistance due to point mutation in the coagulation factor V gene [16]. Prothrombin G20210A mutation causes a G to A transition at the nucleotide position 20210, leading to the production of hyperactive prothrombin and an increase in its levels. MTHFR acts on homocysteine metabolism by reducing 5, 10-methylenetetrahydrofolate to 5-methylenetetrahydrofolate. Thus, enzyme polymorphisms and low folate levels cause a reduction in enzyme activity, resulting in hyperhomocysteinemia, which is a risk factor for thrombosis [17, 18]. Concerning epigenetic mechanisms, the COVID-19 viruses use these mechanisms and increase its severity by affecting host antigen presentation through DNA methylation and histone modifications causing interfere with innate and adaptive immunity, adequacy of inflammatory response, and outcome of viral infections [19]. Regarding to coagulation, citrullination which is a particular histone modification and some miRNAs are associated with venous thromboembolism. No other studies was found on DNA methylation in thrombosis. Also, no validated epigenetics biomarkers are routinely used for diagnosis and prevention of venous thromboembolism [20]. That’s why our study focused on genetic factors only.

We aimed to determine the relationship between the thrombophilia gene mutations (prothrombin G20210A, factor V Leiden, and MTHFR gene) and the severity of COVID-19 patients.

## Materials and methods

### Study population

**Fig 1:**
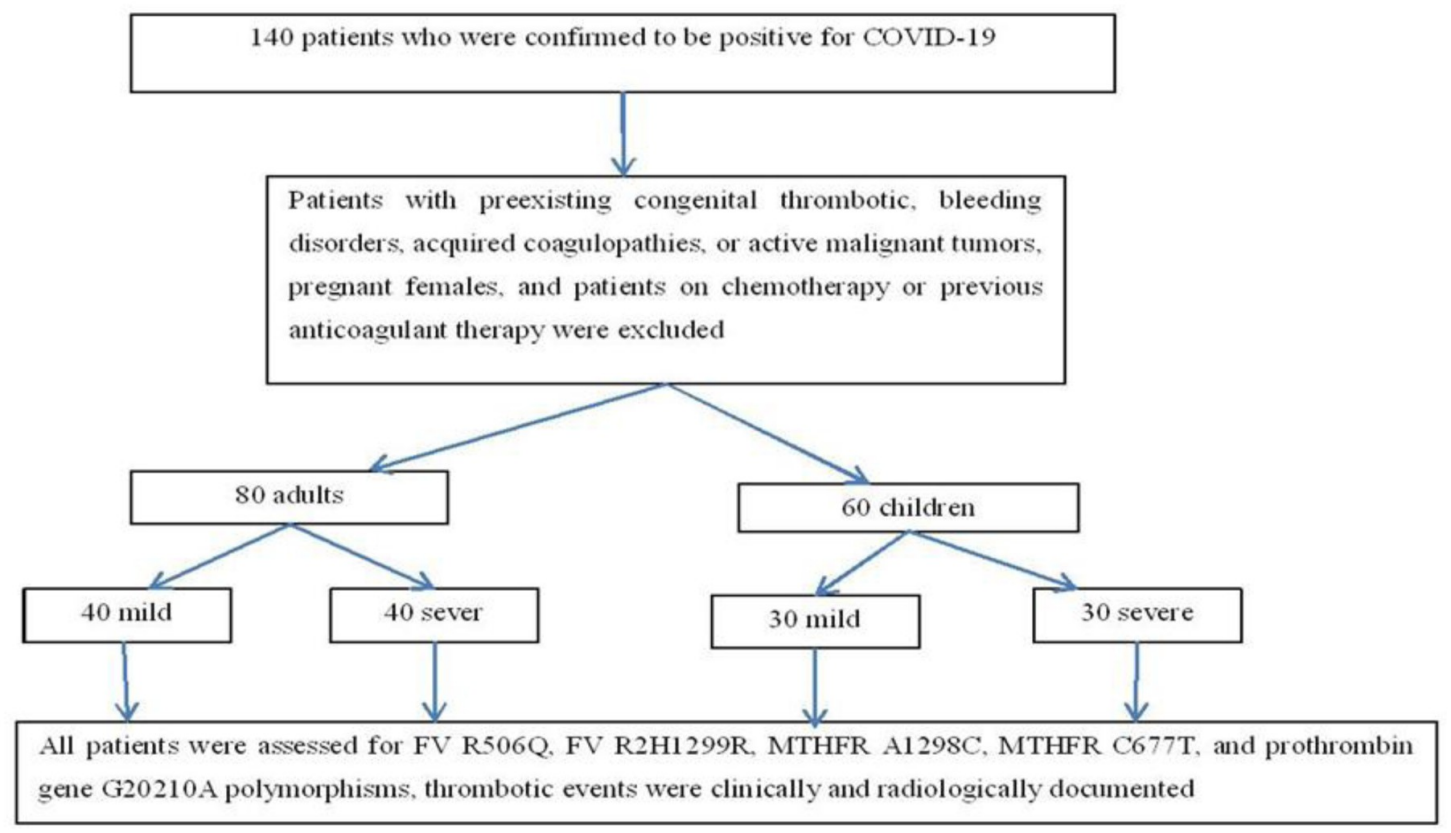
Flowchart of the study. One hundred and forty positive COVID 19 patients were tested for the most popular thrombophilic genes.

This prospective cross-sectional study was carried out at Minia University Hospitals from January 2021 to March 2021. Because there were no previous studies recorded for prevalence of covid-19 and its severity during the time of study, we selected all patients admitted at Minia University Hospitals which included one hundred and forty patients, who were confirmed to be positive for COVID-19 by nasal swabs. Moreover, those patients did not receive any vaccines for COVID-19 because at the time of the study vaccination was not completely applied to all populations. Patients participating in the study were 80 adults and 60 children. They were divided into 70 severe COVID-19 patients admitted to either intensive care units (ICU) or pediatric intensive care units (PICU) (40 adults and 30 children) and 70 mild COVID-19 patients (40 adults and 30 children). Mild COVID-19 cases were recruited from the triage area (classification of mild and severe cases according to WHO guidelines 2021). The current study was conducted according to the ethical guidelines of both the Declaration of Helsinki and the International Conference on Harmonization Good Clinical Practice. In addition, written informed consent was obtained from each patient or first-degree relative. The study was approved by the Institutional Review Board and Medical Ethics Committee of Minia University (Approval No. 706:12/2020). Patients with preexisting congenital thrombotic or bleeding disorders, acquired coagulopathies, or active malignant tumors, pregnant females, and patients on chemotherapy or previous anticoagulant therapy were excluded, Fig 1.

All participating patients were subjected to thorough clinical examination. Laboratory and radiological imaging were performed according to the Egyptian Ministry of Health COVID-19 guidelines and according to their clinical conditions [21]. Mild cases were either asymptomatic or symptomatic (upper respiratory tract illness with or without one of the following symptoms: fever <38, cough, gastrointestinal symptoms, myalgia, or arthralgia) with leucopenia or lymphopenia with no radiological evidence of pneumonia [22]. Severe COVID-19 was defined as the presence of radiological evidence of more than 50% lung infiltrate plus one of the following: respiratory rate of ≥30 breaths per minute; oxygen saturation (SaO2) <94% while breathing room air at rest; or ARDS defined as arterial oxygen partial pressure (PaO2) to a fraction of inspired oxygen (FiO2) (PaO2: FiO2) of ≤30 mmHg. Severe COVID-19 was diagnosed by respiratory failure requiring ventilator support or septic shock, with or without organ dysfunction requiring supportive treatment in ICU [23].

### Laboratory investigations

Eight ml of venous blood samples was withdrawn from all patients. The sample was divided into (1) 1 ml in sterile vacutainers tube containing EDTA solutions for CBC assay; (2) 2 ml in a sterile vacutainer tube containing EDTA solutions for DNA isolation and further assessment of genetic detection; (3) 1.8 ml of blood on a sterile vacutainer tube containing 0.2 ml trisodium citrate for measuring D-dimer; (4) 3 ml of venous blood put into serum separator gel vacutainer tube, with the sample being allowed to clot for 30 minutes at 37°C before centrifugation for 15 minutes at 3,500 rpm. The expressed serum was utilized for assessing the renal and liver function tests, hs-CRP, ferritin, and LDH.

CBC was performed by automated cell counter Celltac ES (Nihon Kohden Europe). D-dimer and hs-CRP were assessed by specific protein analyzer using nephelometry method (Shenzhen Genius Electronics, China). Renal and liver function tests were measured by the automated chemistry auto-analyzer system (Selectra proM, ELITech Group, Finland). Serum ferritin was assessed via the enzyme-linked fluorescent assay (ELFA) technique (Mini Vidas, Biomerieux, France). Finally, LDH activity was assayed using Sigma-Aldrich kits.

Concerning genetic analysis, patients’ genomic DNA was extracted from peripheral blood samples on EDTA (QiaAmp DNA extraction kit, Qiagen). Then, an evaluation of the extracted DNA quantity was carried out using a Nanodrop ND-2000 spectrophotometer (Thermofisher Scientific, USA). FV Leiden, MTHFR, and prothrombin gene mutations were analyzed in all patients using reversed hybridization assay strips obtained from VIENNA LAB GmBH (Vienna, Austria). First, these genes are amplified by polymerase chain reaction. Then, the amplicons were explicitly hybridized to a test strip containing allele-specific oligonucleotide (corresponding to wild type or mutant). A positive reaction was detected by purple staining on a strip. For each polymorphic position, three patterns were obtained: homozygous normal, heterozygous, or homozygous mutant genotype. The patients were assessed for FV R506 Q, FV R2H1299R, MTHFR A1298C, MTHFR C677T, and prothrombin gene G20210A polymorphisms.

### Imaging studies

#### Color duplex study

Color duplex is a rapid, easy applicable, and available imaging tool for examining the state of most body vessels, mainly those peripheral vessels at the upper and lower limbs. It is also utilized for detecting carotid vessels and main mesenteric vessels.

#### MDCT of the brain without contrast

MDCT of the brain without contrast is utilized for detecting cerebral infarction.

#### MDCT angiography of the abdominal vessels

MDCT angiography of the abdominal vessels is a second-line imaging to detect the state of deep vessels, mainly those subbranches at the abdomen and cerebral circulation.

#### MDCT of the abdomen with contrast

MDCT of the abdomen with contrast is used to detect solid organ infarction, such as splenic infarction.

#### Brain MRI DWI

Brain MRI DWI is utilized for the detection of undetectable early ischemic changes in the brain.

In cases with cerebral stroke, MDCT of the brain showed ill-defined hypodense areas, wedge in shape, cortical, and subcortical in location, and others were periventricular in location. For clinically suspected infarction with no CT brain abnormality, MRI brain diffusion-weighted image (DWI) was diagnostic at small and early brain affection, where restricted diffusion appears at the affected brain areas. In cases with suspected mesenteric vascular occlusion, MDCT angiography of the abdominal vessels showed non-enhanced occluded superior mesenteric artery, and other cases showed dilated small bowel loops with paper-thin wall and air density within the wall denoting pneumatosis intestinalis. In cases with lower limb ischemia, the color duplex of lower limb arteries showed occluded segments of the affected arteries with loss of color flow at the affected segments.

### Statistical analysis

Statistical analysis was conducted using SPSS software version 18.0 (SPSS Inc., Chicago, IL, USA). Quantitative variables were reported as mean ± standard deviation (SD), whereas categorical variables were reported as numbers and percentages. Pearson’s chi-square (*χ*2) and Fisher’s exact tests were utilized for assessing the genotype significance and allele frequencies in COVID-19 patients by comparing mild and severe cases. Then, the odds ratio and 95% confidence interval were calculated for each gene polymorphism. Homozygote mutant and heterozygote genotypes of each group were brought together as a new group. Next, odds ratios and 95% confidence intervals using logistic regression analysis were calculated. *P* values less than 0.05 were considered statistically significant.

## Results and discussion

### Demographic and laboratory data of the studied subjects

The demographic and laboratory data of the studied subjects are presented in Table 1. Patients in the severe COVID-19 group had significantly higher hs-CRP, D-dimer, LDH, and ferritin in comparison with patients in the mild COVID-19 group (p<0.05 in all).

**Table 1:** Demographic and laboratory data of the studied COVID-19 patients.

Data	Child			Adult		
	Severe n = 30	Mild n = 30	p	Severe n = 40	Mild n = 40	p
**Age (years): mean**±SD	2.5±1.3	2.6±1.2	0.9	33±8.3	34.5±7.7	0.5
**Sex**						
Male: n (%)	13(86.7%)	10(66.7%)	0.1	8(60%)	12(60%)	0.2
Female: n (%)	2(13.3%)	5(33.3%)		12(40%)	8(40%)	
**Renal function**						
Urea (mg/dl): **mean**±SD	53.7±36.9	33.2±11.1	0.04*	37.8±5.5	37.8±5.6	0.9
Creatinine (mg/dl): **mean**±SD	0.78±0.22	0.77±0.23	0.9	1.08±0.1	1.07±0.1	0.9
**Liver function**						
AST (U/l): **mean**±SD	43.8±12.1	39±5.1	0.1	32.4±2.8	32.4±2.69	0.9
ALT (U/l: **mean**±SD	42.9±12.8	39±8.2	0.3	44±2.3	43.6±1.9	0.5
Albumin (g/dl): **mean**±SD	3.9±0.35	3.9±0.33	0.9	4.3±0.3	4.3±0.3	0.9
Total bilirubin (mg/dl): **mean**±SD	0.84±0.09	0.84±0.09	0.9	0.87±0.08	0.86±0.08	0.9
**Hb (gm/dl): mean**±SD	11.3±2.1	11.5±1.4	0.7	11.4±2.2	11.2±3.2	0.7
**PLTs (x10**^**3**^**/cmm): mean**±SD	262.8±243.8	292.8±156.5	0.4	307.2±151.1	283.4±109.6	0.5
**WBCs (x10**^**3**^**/cmm): mean**±SD	16.2±7.2	13.5±5.8	0.6	17.2±3.1	16.6±3.2	0.5
**Lymphocyte (%): mean**±SD	17.2±5.4	19.7±4.4	0.1	11±3.1	11.1±4.4	0.6
**Lymphocyte (x10**^**3**^**/cmm): mean**±SD	2886.1±1720.1	3595.8±1435.1	0.2	1756±477.7	1952±671.1	0.2
**Neutrophil (%): mean**±SD	71.8±4.8	73.1±3.5	0.5	78.6±2.4	78.9±2.3	0.6
**hs CRP** (mg/dl): **mean**±SD	104.1±16.04	11.3±4.4	0.001^a^	98.1±7.7	11.1±2.6	0.001^a^
**D-dimer** (μg/ml): **mean**±SD	1.4±1.7	0.30±0.20	0.02 ^a^	1.7±1.2	0.37±0.27	0.001 ^a^
**Ferritin (ng/ml): mean**±SD	658±534.1	199.9±50.4	0.003 ^a^	564±73.8	246.2±25.1	0.001 ^a^
**LDH** (U/l): **mean**±SD	608±94.6	393.3±72.8	0.0001 ^a^	611.5±96.5	342.2±94.1	0.0001 ^a^

AST: aspartate transaminase, ALT: alanine transaminase, Hb: hemoglobin, PLTs: platelets, WBCs: white blood cells, hs-CRP: high sensitive C-reactive protein, and LDH: lactic dehydrogenase enzyme.

^a^ Statistical significance p<0.05.

#### Frequency of thrombotic complications among severe cases

**Fig 2:**
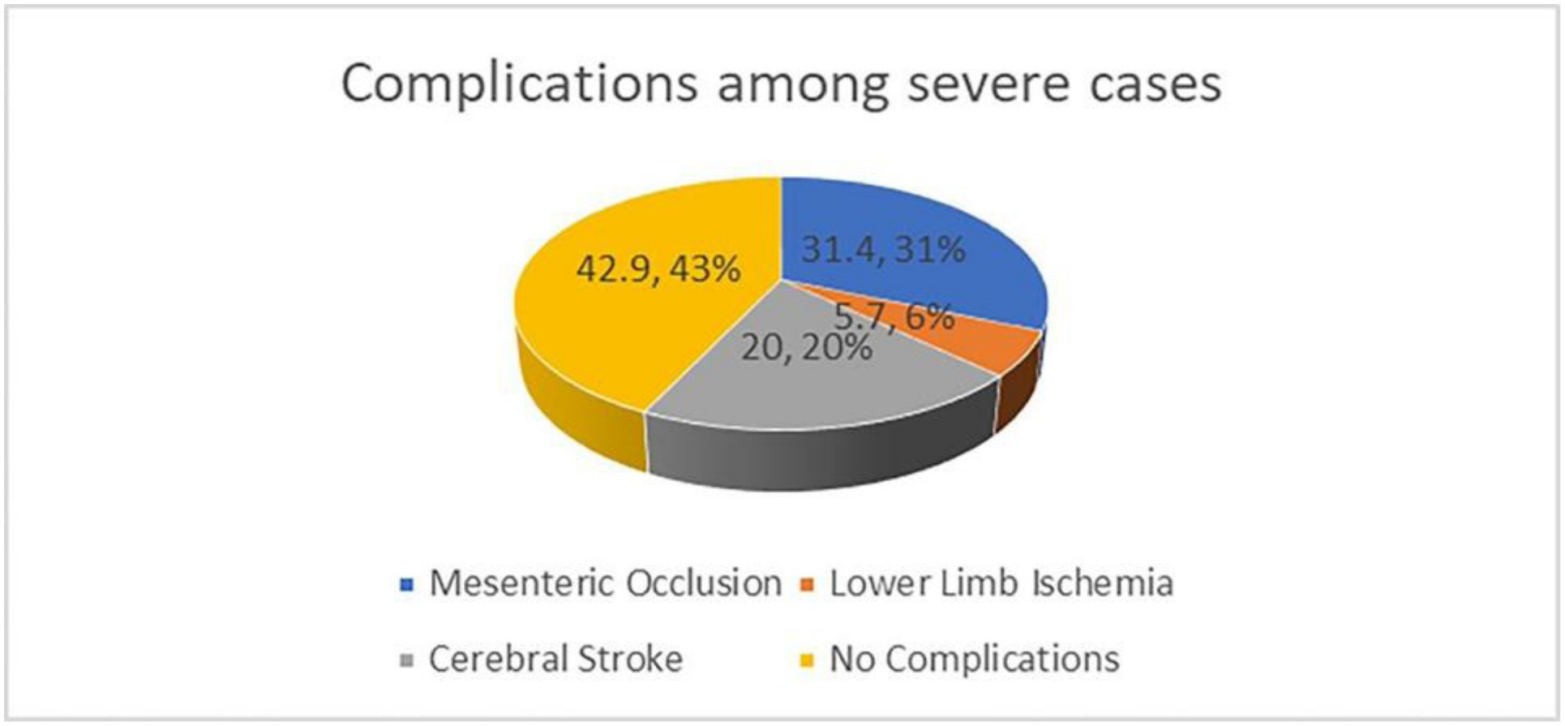
Mesenteric occlusion 22/70 (31.4%), lower limb ischemia 4/70 (5.7%), cerebral stroke 14/70 (20%), and no complications 42.9%.

**Fig 3:**
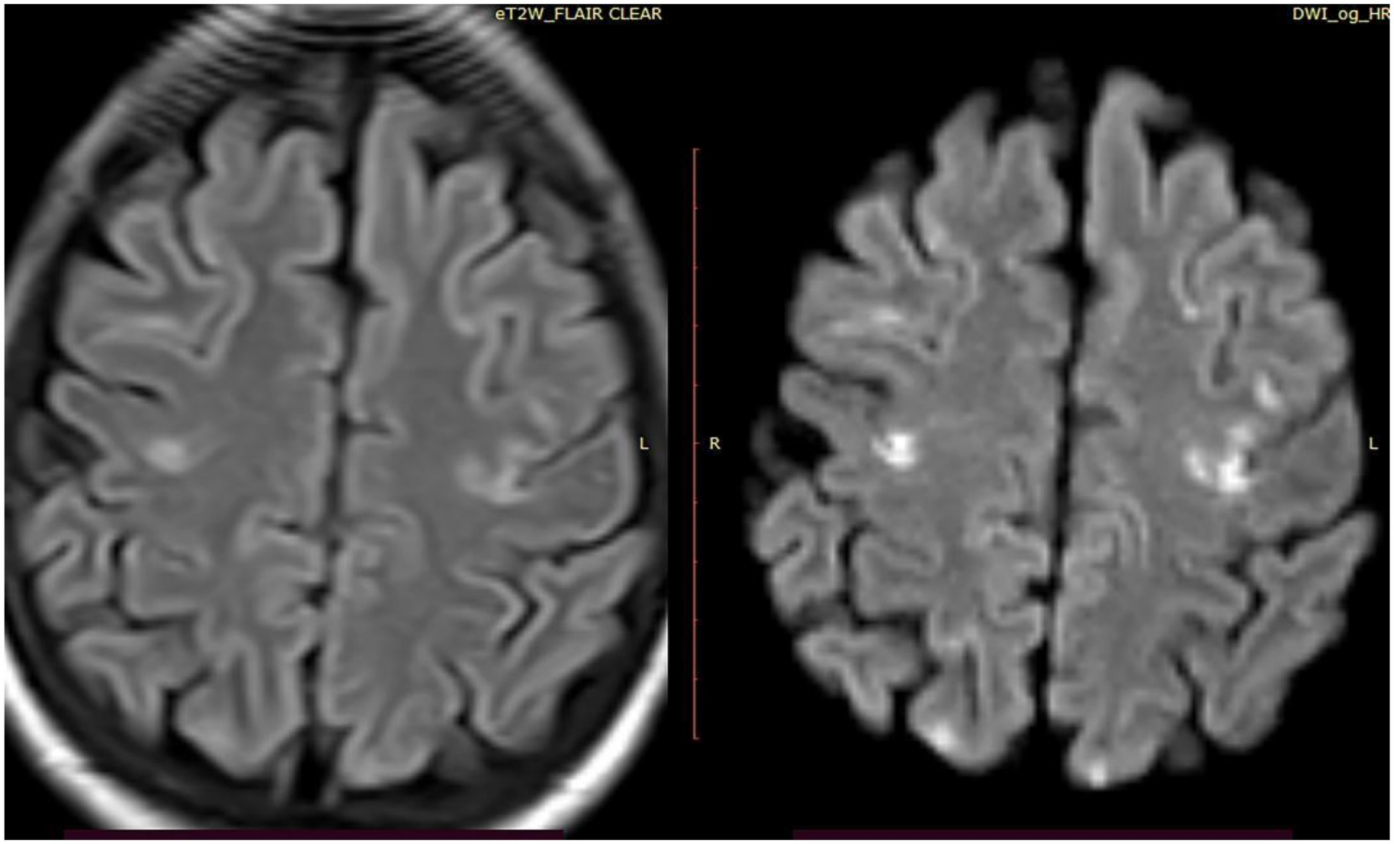
CT and MRI of the brain showed few scattered abnormal signal intensity foci seen at both high parietal regions attaining high signal intensity at FLAIR with true diffusion restriction at DWI.

Regarding thrombotic complications in forty studied severe COVID-19 adult cases, there were 22 (31.4%) cases with mesenteric vascular occlusion and 4 (5.7%) cases with lower limb ischemia. In comparison, there were 14 children (20%) with ischemic stroke representing the thrombotic complication occurring in our pediatric studied population with severe COVID-19, Figs 2 and 3.

#### Frequencies of studied genotypes in adult COVID-19 patients

Regarding the studied genotypes in adult cases, severe COVID-19 cases were significantly more frequent to have a heterozygous mutation for all the studied genes compared to mild COVID-19 cases, and mild cases were significantly more frequent to have normal variants of the studied thrombophilia genes (p<0.05 in all). Moreover, being mutant to gene FV R506 Q carried the highest risk of developing a severe disease course, as the odds ratio was 27 (CI 95%: 7.6–94.8, p<0.0001), Table 2.

**Table 2:** Studied genotypes frequencies in adult patients with mild and severe COVID-19.

Groups Genes	Mild COVID-19 No. = 40 n (%)			Severe COVID-19 No. = 40 n (%)			p value	Odds ratio (95% CI) p value	
	Homozygous normal	Heterozygous	Homozygous mutant	Homozygous normal	Heterozygous	Homozygous mutant		Homozygous normal	Mutant genotypes
**FV R506 Q**	GG	GA	AA	GG	GA	AA	0.0001 ^a^	Reference	27 (7.6–94.8) 0.0001 ^a^
	36 (90%)	4 (10%)	0	10 (25%)	30 (75%)	0			
**FV R2 H1299R**	AA	AG	GG	AA	AG	GG	0.0001 ^a^	Reference	7 (2.6–18.7) 0.0001 ^a^
	30 (75%)	10 (25%)	0	12 (30%)	28 (70%)	0			
**MTHFR** **A1298C**	AA	AC	CC	AA	AC	CC	0.0001 ^a^	Reference	6.8 (2.5–18.1) 0.0001 ^a^
	32 (80%)	8 (20%)	0	14 (35%)	22 (55%)	4 (10%)			
**MTHFR** **C677T**	CC	CT	TT	CC	CT	TT	0.0001 ^a^	Reference	12 (4.1–34.4) 0.0001 ^a^
	32 (80%)	8 (20%)	0	10 (25%)	30 (75%)	0			
**Prothrombin gene** **G20210A**	GG	GA	AA	GG	GA	AA	0.003 ^a^	Reference	8.5 (2.9–24.8) 0.0001 ^a^
	34 (85%)	6 (15%)	0	16 (40%)	24 (60%)	0			

FV: factor V Leiden and MTHFR: methylenetetrahydrofolate reductase.

^a^ Statistical significance p<0.05.

#### Frequencies of studied genotypes in pediatric COVID-19 patients

**Fig 4:**
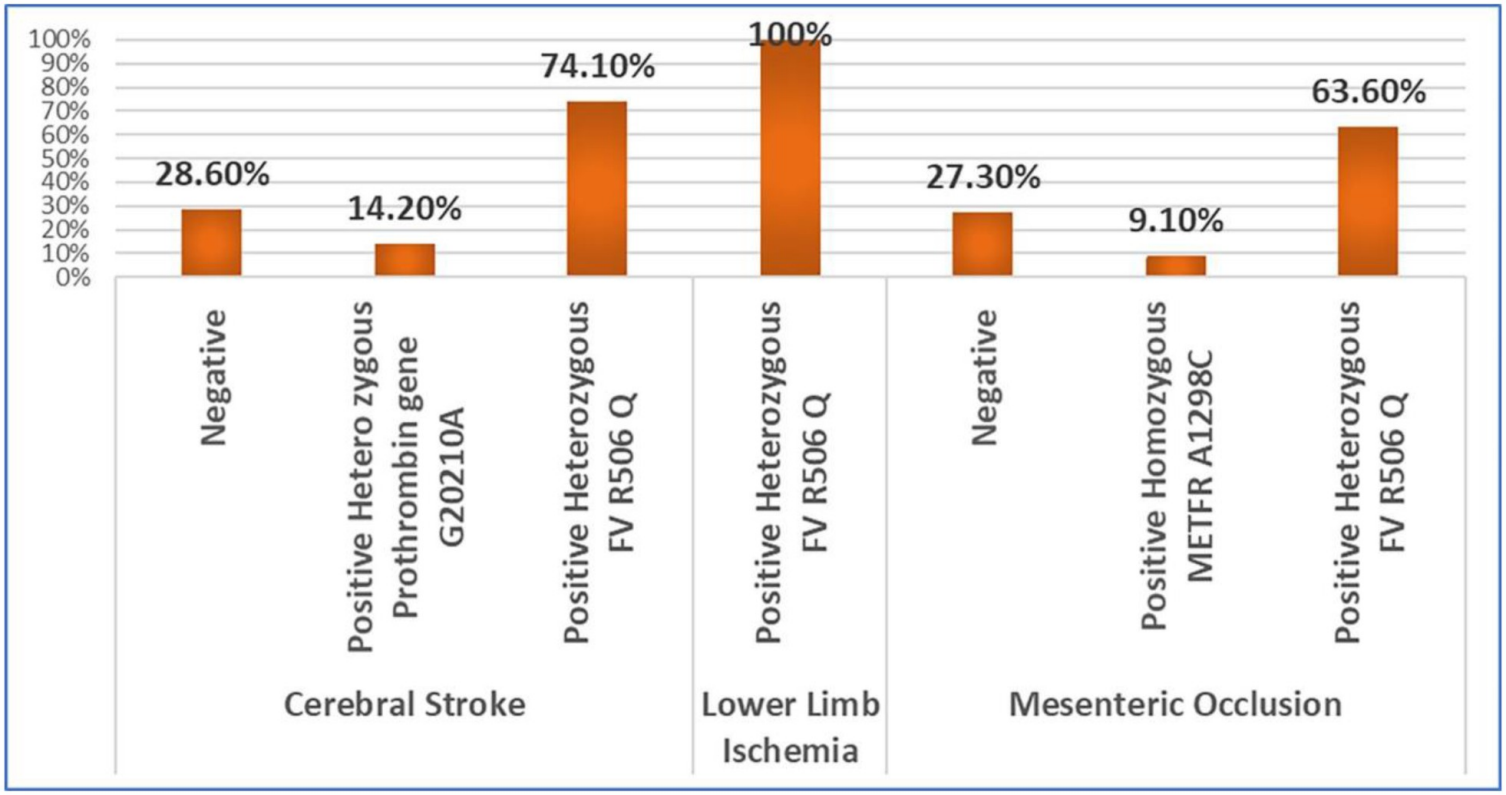
Genotypes of children and adults who developed thrombotic complications.

Like adult COVID-19 patients, pediatric patients with severe COVID-19 were significantly more frequent to have a heterozygous mutation for all the studied genes compared to mild COVID-19 patients, and being a normal variant was more frequently associated with contracting a mild disease course (p<0.05). Being mutant to gene FV R506 Q carried the highest risk of developing a severe disease course, as the odds ratio was 38.5 (CI 95%: 7.4–199.8, p = 0.0001), Table 3. This may explain why eight out of the twelve children who suffered from cerebral stroke were heterozygous for FV R506 Q. Meanwhile, the remaining four children with cerebral stroke were negative for all the studied genes, Fig 4.

**Table 3:** Studied genotypes frequencies in pediatric patients with mild and severe COVID-19.

Groups	Mild COVID-19 No. = 30 n (%)			Severe COVID-19 No. = 30 n (%)			p value	Odds ratio (95% CI) p value	
Genes	Homozygous normal	Heterozygous	Homozygous mutant	Homozygous normal	Heterozygous	Homozygous mutant		Homozygous normal	Mutant genotypes
**FV R506 Q**	GG	GA	AA	GG	GA	AA	0.0001 ^a^	Reference	38.5 (7.4–199.8) 0.0001 ^a^
	28 (93.3%)	2 (6.7%)	0	8 (26.7%)	22 (73.3%)	0			
**FV R2 H1299R**	AA	AG	GG	AA	AG	GG	0.002 ^a^	Reference	6 (1.8–19.04) 0.002 ^a^
	24 (80%)	6 (20%)	0	12 (40%)	18 (60%)	0			
**MTHFR A1298C**	AA	AC	CC	AA	AC	CC	0.001 ^a^	Reference	9.01 (2.5–31.7) 0.001 ^a^
	26 (86.7%)	4 (13.3%)	0	12 (40%)	16 (53.3%)	2 (6.7%)			
**MTHFR C677T**	CC	CT	TT	CC	CT	TT	0.0001 ^a^	Reference	8 (2.4–25.8) 0.001 ^a^
	24 (80%)	6 (20%)	0	10 (33.3%)	20 (66.7%)	0			
**Prothrombin gene** **G20210A**	GG	GA	AA	GG	GA	AA	0.0001 ^a^	Reference	13 (3.5–47.5) 0.0001 ^a^
	26 (86.7%)	4 (13.3%)	0	10 (33.3%)	20 (66.7%)	0			

FV: factor V Leiden and MTHFR: methylenetetrahydrofolate reductase.

^a^ Statistical significance p<0.05.

The increased risk of FV R506 Q mutation among adults and children was evident when we studied the genotype of the complicated cases. Fourteen out of twenty-two patients with mesenteric vascular occlusion were heterozygous for FV R506 Q, and two were homozygous mutant for METFR A1298C. Meanwhile, the remaining six cases with mesenteric vascular occlusion were negative for the studied genes. Furthermore, patients with lower limb ischemia were heterozygous for FV R506 Q, and the cerebral stroke patient was heterozygous for both FV R506 Q and prothrombin gene G20210A. Thus, 22 out of the 28 cases with thrombotic complications were heterozygous for FV R506 Q. The genotypes of children and adults who developed thrombotic complications are presented in Fig 4.

#### Relation of D-dimer to the genotype of the studied COVID-19 patients

When we compared the genotypes of all studied cases according to their D-dimer levels, patients with abnormally high D-dimer levels were significantly more frequent to be heterozygous for FV R506 Q, FV R2H1299R, MTHFR C677T, and prothrombin gene G20210A (p = 0.0001, 0.0001, 0.005, and 0.001, respectively). Furthermore, adults or children, who were heteromutant to the two studied factor V Leiden genes, had the highest risk of hypercoagulability with odds ratios of 4.1 (CI 95%: 1.4–11.4, p = 0.007) and 3.9 (CI 95%: 1.4–10.7, p = 0.008) for FV R506 Q and FV R2H1299R, respectively, Table 4.

**Table 4:** Relation of D-dimer to the genotype of the studied COVID-19 patients.

Data	Normal D-dimer No. = 66 n (%)	High D-dimer No. = 74 n (%)	P	Odds ratio	95% CI of OR	p
**FV R506 Q**						
**GG**	50 (75.8%)	32(43.2%)		Reference	Reference	
**GA**	16 (24.2%)	42(56.8%)	0.0001 ^a^	4.1	1.9–8.4	0.0001 ^a^
**AA**	0	0				
**FV R2 H1299R**						
**AA**	48 (72.7%)	30(40.5%)		Reference	Reference	
**AG**	16 (27.3%)	44(59.5%)	0.0001 ^a^	3.9	1.9–7.9	0.0001 ^a^
**GG**	0	0				
**MTHFR A1298C**						
**AA**	46 (69.7%)	38(51.4%)		Reference	Reference	
**AC**	18 (27.3%)	32(43.2%)	0.08	1.9	1.04–3.5	0.03 ^a^
**CC**	2 (3%)	4 (5.4%)				
**MTHFR C677T**						
**CC**	44 (66.7%)	32(43.2%)		Reference	Reference	
**CT**	22 (33.3%)	42(56.8%)	0.005 ^a^	2.6	1.3–5.2	0.006 ^a^
**TT**	0	0		-		-
**Prothrombin gene G20210A**						
**GG**	50 (75.8%)	36(48.6%)		Reference	Reference	
**GA**	16 (24.2%)	38(51.4%)	0.001 ^a^	3.2	1.5–6.8	0.001 ^a^
**AA**	0	0		-		-

FV: factor V Leiden and MTHFR: methylenetetrahydrofolate reductase.

^a^ Statistical significance p<0.05.

COVID-19 was noticed to be associated with both venous and arterial thromboembolic disease due to severe inflammation, hypoxia, and diffuse intravascular coagulation (DIC) [24]. It was recorded that 31% of ICU patients with COVID-19 had thrombotic complications [6]. During the COVID-19 pandemic, many Egyptian patients presented to the emergency unit with thrombotic complications such as mesenteric vascular occlusion, lower limb ischemia, and cerebral strokes. Most of these patients had a history of COVID-19 infection. A complex collaboration among various factors triggers the susceptibility to thrombosis in COVID-19 patients, as thrombosis is often associated with severe cases and is related to poor prognosis. Furthermore, congenital thrombophilia is associated with early and recurrent thrombosis and increased risk of thrombosis, particularly if combined with additional factors [25]. Factor V Leiden accounts for 40–50% of thrombosis, and prothrombin G20210A is considered the second most common cause of genetic mutation causing thrombophilia [26, 27]. Moreover, methylene tetrahydrofolate reductase (MTHFR) plays a role in folate metabolism and disturbed function of this enzyme causing hyperhomocysteinemia and thrombophilia [27, 28]. Thus, our study aimed to determine the relationship between the severity of COVID-19 and the presence of these common thrombophilia gene mutations (prothrombin gene, factor V Leiden, and MTHFR gene) in COVID-19 Egyptian patients. In the current study, adults and children with severe COVID-19 were significantly more frequent to have a heterozygous mutation for all the studied genes compared to mild COVID-19 cases, and being a normal variant was more frequently associated with contracting a mild disease course. This agreed with the few studies addressing the relationship between thrombophilia genes and COVID-19 disease course. The C677T polymorphism of MTHFR had been associated with susceptibility to develop a severe course of coronavirus disease 2019 (COVID-19) initiated by hyperhomocysteinemia [29]. In addition, increased activity in factor V had been reported in patients with severe disease [30, 31]. The relationship between digital vein thrombosis in COVID-19 patients and factor V Leiden mutation carrier revealed marked inflammation triggered by the virus and consequent endothelial dysfunction and thrombosis [32]. Similarly, prothrombin G20210A gene mutation was also found to be associated with an increased thrombotic tendency [33] and thrombotic complications in COVID-19 patients [34]. That agreed with a case study of a 31-year-old Caucasian patient with COVID-19 who suffered from persistent abdominal and back pain. The abdominal CT revealed thromboembolism in the main branches of the pulmonary artery, tributary branch for the lower left lobe, lower and middle right lobes, and common left iliac vein. It was reported that this patient had mutations in factor V Leiden (FV R506 Q) and the prothrombin gene (G20210A) along with the presence of anti-phospholipid antibodies [35].

Another case of a 48-year-old male patient presented with thrombosis in ventricles, pulmonary arteries, and peripheral vein. His genetic study of thrombophilia revealed heterozygous mutations for factor V Leiden, prothrombin, and plasminogen activator inhibitor PAI-1 [36]. The study findings of Kiraz, Aslihan et al. were against our findings. They ruled out the relationship between common thrombophilia genes SNPs of factor II, factor V, and PAI-1 [37]. The contradiction may be explained by the fact that they did not compare the genotypes of severe COVID-19 patients with those of mild COVID-19 patients. Instead, they compared those with the genotypes of healthy individuals from the pre-COVID-19 era.

Being mutant to gene FV R506 Q in this study carried the highest risk of developing a severe disease course. Furthermore, eleven of the fourteen adult cases who suffered from thrombotic complications and four of the six children who suffered from cerebral stroke were heterozygous for FV R506 Q. Increased factor V and factor VIII activity were also linked to the increased thrombotic tendency [38]. Additionally, it was discovered that megakaryocytes are abundant in the lungs, heart, and other organs of COVID-19 patients [39]. Since megakaryocytes produce platelets, which generally contain about 20–25% of factor V in blood, this might be related to the mechanism for the high factor V found in severe COVID-19 cases [30]. Dysregulation of factor V due to factor V Leiden is a well-known cause of a prothrombotic state [40]. Additional another variant in exon 13 of the factor V gene, an A change to G at nucleotide 4070 results in His to Arg amino acid substitution at position 1299 this known as R2 or H1299R [41]. FV H1299R was known to related to hereditary thrombophilia, several studies documented that this mutation increase venous thrombosis by 2 to 3 folds [42]. Gustavo Cernera et al study that investigated the role of multiple thrombotic genes in a patients with different venous thromboembolic diseases, showed that factor V, factor V R2 and FII G20210A were related to thrombosis [43]. Moreover Ivana Lapić et al research revealed that severity of COVID 19 course related to heterogenicity of both FV H1299R and FXIII V34L polymorphisms [44]. These findings designate factor V as a possible biomarker of COVID-19 thrombotic tendency with potential links to SARS-CoV-2 disease biology [30].

In the present study, adults and children with severe COVID-19 were more frequently to be heteromutant to the MTHFR polymorphisms. One patient who developed mesenteric vascular occlusion was a homozygous mutant for MTHFR A1298C. This may be attributed to the relationship between MTHFR polymorphisms and reduced glutathione levels as the folate cycle, the methionine cycle, and the trans-sulfuration pathway are linked together. S-adenosyl-methionine levels are lowered in states of low MTHFR activity, resulting in decreased glutathione synthesis [45]. It was found that therapeutic supplementation of glutathione resulted in a rapid symptom improvement in two COVID-19 cases, all of which point to the prominent role of glutathione in the anti-oxidative defense system in viral illness [46].

Moreover, our study demonstrated that patients with heterozygous genotypes of METFR C677T and A1298C were highly susceptible to thrombosis in severe cases. The MTHFR enzyme converts 5, 10-methylenetetrahydrofolate to 5-methyltetrahydrofolate responsible for conversion of homocysteine to methionine. The SARS-CoV-2 remodels both the host folate and the one-carbon metabolism at the post-transcriptional level in order to meet the demand for viral RNA replication. Therefore, folate decreased in SARS-CoV-2- infected cells, and homocysteine levels increased which become risk factors for cardiovascular and neurological diseases and severe course of COVID-19 [33, 34]. Therefore, MTHFR variants and homocysteine levels have been considered the modulators of COVID-19 incidence and severity [47, 48]. Moreover, homocysteine regulatory mechanisms activate the angiotensin II type receptors [49, 50]. SARS-CoV-2 enters and infects cells through angiotensin II receptor, causing changes in DNA methylation which may result in increased susceptibility to SARS-CoV-2 [29].

A correlation analysis was carried out by Ponti G et al. and recorded a strong correlation between C 677T allele and death from coronavirus with 85% [47]. Recently, the correlation between homocysteine level and COVID-19 severity has been demonstrated as hyperhomocysteinemia (>15.4 μmol/L) had a three-fold increased risk of progression CT change [50, 51]. Regarding METFR C677T, the world prevalence of both the CT and TT genotype was found to be increased among Europeans (54.0%) and North Americans (42.8%) and decreased in Asians (35.4%) and Africans (19.6%). However, in some East Asian countries, both genotypes were found to have the highest prevalence in China (67.1%) and the lowest in India (20.3%). In European countries, the highest prevalence was detected in Italy (66.3%) and the lowest in Finland (44.2%) [52, 53]. These data can explain the highest mortality among the Italian and Chinese populations. The present study recorded that the frequency of CT genotype was 75% and 66.7% among adults and children, respectively, but no TT genotype was detected.

When we compared the genotypes of all studied cases according to their D-dimer level, patients with abnormally high D-dimer levels were significantly more frequent to be heterozygous for FV R506 Q, FV R2H1299R, and prothrombin gene G20210A. This agreed with de la Morena‐Barrio et al. [34]. Their pooled analysis revealed higher D-dimer levels in the presence of inherited thrombophilia than in its absence. They attributed that to the defective control of thrombin generation occurring in congenital thrombophilia that may be exacerbated in stress situations such as COVID-19 infection [34]. Numerous studies reported that increased in-hospital mortality was associated with increased D-dimer levels, suggesting the usefulness of D-dimer as a biomarker for clinical outcomes in COVID-19 patients [34, 54, 55].

The limitation of the study was that other thrombophilic genetic mutations can be included but unfortunately we couldn’t do this because of low financial support. So, we focused only in most common mutations in our population.

## Conclusions

There is an evident relationship between severe COVID-19 and inherited thrombophilia, patients with thrombophilia had significantly higher D-dimer levels than those without identifiable inherited thrombophilia. FV R506 Q gene mutation in this study carried the highest risk of developing a severe COVID-19 course. Finally, our study focused on possible complications and their relation to genetic mutation. Further research is needed to highlight the clinical outcomes of inherited thrombophilia states in SARS-CoV-2 infection and guide the optimal anti-coagulation management in these cases.

## Supporting information

S1 File All data results of study. (XLS)
